# *PGC1α* −1 Nucleosome Position and Splice Variant Expression and Cardiovascular Disease Risk in Overweight and Obese Individuals

**DOI:** 10.1155/2014/895734

**Published:** 2014-12-28

**Authors:** Tara M. Henagan, Laura K. Stewart, Laura A. Forney, Lauren M. Sparks, Neil Johannsen, Timothy S. Church

**Affiliations:** ^1^Department of Nutrition Science, Purdue University, 700 W. State Street, West Lafayette, IN 47907, USA; ^2^Rocky Mountain Cancer Rehabilitation Institute, University of Northern Colorado, Greeley, CO 80639, USA; ^3^School of Kinesiology, Louisiana State University, Baton Rouge, LA 70803, USA; ^4^Florida Hospital Sanford-Burnham Translational Research Institute for Metabolism and Diabetes, Orlando, FL 32804, USA; ^5^Laboratory of Preventative Medicine, Pennington Biomedical Research Center, Baton Rouge, LA 70808, USA

## Abstract

*PGC1α*, a transcriptional coactivator, interacts with PPARs and others to regulate skeletal muscle metabolism.* PGC1α* undergoes splicing to produce several mRNA variants, with the* NTPGC1α* variant having a similar biological function to the full length* PGC1α* (*FLPGC1α*). CVD is associated with obesity and T2D and a lower percentage of type 1 oxidative fibers and impaired mitochondrial function in skeletal muscle, characteristics determined by* PGC1α* expression.* PGC1α* expression is epigenetically regulated in skeletal muscle to determine mitochondrial adaptations, and epigenetic modifications may regulate mRNA splicing. We report in this paper that skeletal muscle* PGC1α*  −1 nucleosome (−1N) position is associated with splice variant* NTPGC1α* but not* FLPGC1α* expression. Division of participants based on the −1N position revealed that those individuals with a −1N phased further upstream from the transcriptional start site (UP) expressed lower levels of* NTPGC1α* than those with the −1N more proximal to TSS (DN). UP showed an increase in body fat percentage and serum total and LDL cholesterol. These findings suggest that the −1N may be a potential epigenetic regulator of* NTPGC1α* splice variant expression, and −1N position and* NTPGC1α* variant expression in skeletal muscle are linked to CVD risk. This trial is registered with clinicaltrials.gov, identifier NCT00458133.

## 1. Introduction

Obesity and type 2 diabetes (T2D), well-known risk factors for cardiovascular disease (CVD), are marked by metabolic disturbances resulting partially from skeletal muscle mitochondrial maladaptations [1–3]. CVD itself is associated with a low percentage of type 1 oxidative skeletal muscle fibers and impaired mitochondrial function in skeletal muscle. Associated maladaptations, including decreases in mitochondrial number and function, are highly dependent on controllable risk factors which have the potential to alter the epigenome [4–6].* Peroxisome proliferator-activated receptor gamma coactivator 1 alpha* (*PGC1α*) is an important regulator of mitochondrial adaption and metabolism in several tissues, including skeletal muscle, due to its transcriptional coactivator function and binding to PPARs, estrogen related receptor alpha (ERR*α*), nuclear receptor factor 1 (Nrf1), and others.* PGC1α* has been recently shown to be epigenetically regulated [7], and its splicing produces the novel, biologically relevant N-terminal truncated mRNA variant (*NTPGC1α*) [8]. In adipocytes,* NTPGC1α* expression acts in a similar manner to the unspliced* PGC1α* variant (*FLPGC1α*) to determine mitochondrial adaptations and compensates for loss or downregulation of* FLPGC1α* [8, 9].

*FLPGC1α* and* NTPGC1α* expression link environmental stimuli to mitochondrial adaptations and metabolism. For example, it has been recently shown that* PGC1α* is hypermethylated via recruitment of DNA methyltransferase 3b (DNMT3b) in T2D muscle and upon treatment with the fatty acids palmitate and oleate [7]. The nucleosome core position determines recruitment of DNMT3b [10] as well as determining the chromatin structure and access of RNA pol II and other transcription factors to the DNA template for transcription to successfully occur [11, 12]. The nucleosome position itself is partially determined by the combinatorial effects of epigenetic modifications in the cell [13] and may serve as an identifier of epigenetically regulated genomic loci in addition to its role in chromatin dynamics and gene regulation. Interestingly, new studies have also suggested a novel role for nucleosomes, particularly the −1 nucleosome (−1N) which is the first nucleosome within the promoter region that is directly upstream of the TSS [8], in determining mRNA splicing and variant expression [11].

Although* NTPGC1α* has been shown to be expressed in other tissues such as skeletal muscle, it is unknown if* NTPGC1α* expression is altered in association with disease risk, if its expression is linked to beneficial metabolic outcomes similar to increased* FLPGC1α* expression, or if* NTPGC1α* expression is epigenetically regulated. For this study, we used a subset of baseline muscle samples from the Health Benefits of Aerobic and Resistance Training in Type 2 Diabetes (HART-D) study [14] to define the relationship between the −1N position in* PGC1α*,* FLPGC1α*, and* NTPGC1α* splice variant expression in skeletal muscle and cardiometabolic risk factors in overweight/obese individuals with T2D.

## 2. Materials and Methods

### 2.1. Participants

Collection and use of skeletal muscle samples from the HART-D study [12, 14] were approved by the Institutional Review Board of the Pennington Biomedical Research Center. The clinical trial has been registered at clinicaltrials.gov, identifier NCT00458133. From a pool of 80 samples, 15 were randomly selected to represent tissue from overweight/obese (BMI > 30) individuals (*n* = 4 female and 11 male), aged 39–67, with T2D. Researchers involved with the analysis of the muscle tissue and resultant data were blinded to any previously obtained data from all participants. Skeletal muscle samples were collected by biopsy and cryopreserved until use in this study as previously reported [12].

### 2.2. Scanning qPCR

Genomic and mononucleosomal DNA, or DNA within one nucleosome, were isolated from 10 mg of skeletal muscle tissue ground under liquid nitrogen as previously described [15]. Briefly, nuclei were extracted from quadriceps muscle in a 0.25 M sucrose buffer (0.25 M sucrose, 10 mM Tris-acetate pH 8.1, 1 mM EDTA, 1 mM DTT, 1 mM sodium orthovanadate, and 1 1X complete protease inhibitor tablet (Roche 11873580001)). After washing, pellets were resuspended in 0.25 sucrose buffer containing 0.1 N CaCl_2_ and 4 mM MgCl_2_ and incubated with micrococcal nuclease (MNase; Roche) for mononucleosomal DNA extraction or without MNase for genomic DNA extraction for 15 min at 37°C. EDTA was added to a final concentration of 5 mM. Pellets were formed by centrifugation and lysed with 0.25 M lysis buffer (50 mM Tris, pH 8.1, 10 mM EDTA, 1% SDS, and 1 1X complete protease inhibitor tablet (Roche 11873580001)). All samples were treated with 0.1 mg/mL of proteinase K (Qiagen) overnight at 37°C to remove histone proteins.

**Figure 1:**
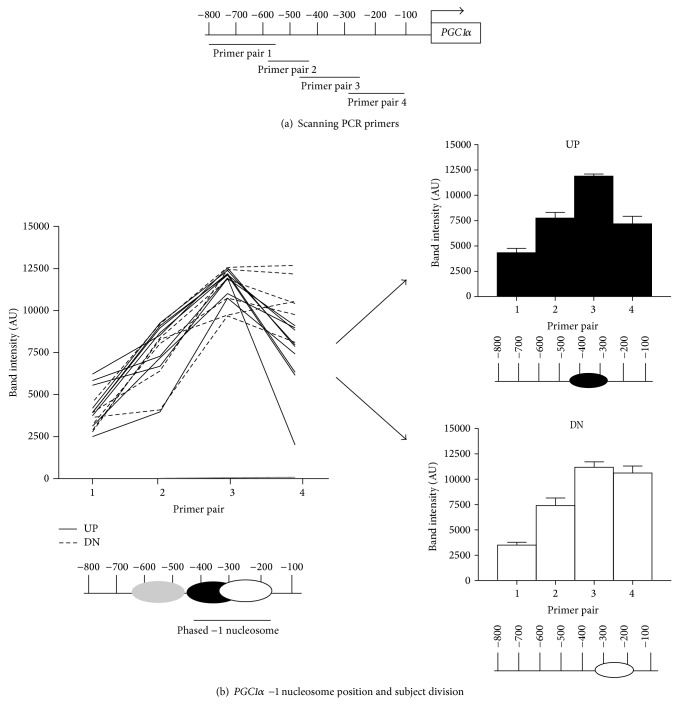
*PGC1α* −1 nucleosome position. (a) Scanning qPCR was performed using four overlapping primer pairs targeted to the* PGC1α* promoter region from approximately the −800 nt to the −100 nt and depicted in the schematic. (b) Nucleosome positions were determined based on densitometry results and plotted as a line graph for each individual. All participants showed similar amplification with primer pairs 1 and 2, depicted as an upstream nucleosome (gray). Similar amplification was also seen with primer pair 3 but not with primer pair 4. This amplification pattern is depicted as a phased −1N positioned between −170 nt and −440 nt (white and black) below the line graph. Based on the phased −1N position, participants were divided into two experimental groups shown on the right: upstream (UP, black) and downstream (DN, white), and densitometry results for each group are shown as mean ± SEM in the bar graphs. −1N for UP and DN is depicted beneath each bar graph. All nucleosome positions are depicted relative to the transcriptional start site (TSS).

Scanning qPCR was performed as previously described [16, 17]. Overlapping primers (sequences presented in Table 1) were designed to cover the* PGC1α* gene promoter region, ranging from ~−800 nucleotide (nt) to the −100 nt (Figure 1). PCR products for both mononucleosomal and genomic DNA samples were run on a 1.5% agarose gel and visualized on a Molecular Imager Gel Doc XR (Biorad, Hercules, CA). Densitometry was performed using MacBiophotonics ImageJ (Bethesda, MD), with mononucleosomal band intensity being divided by the intensity of the corresponding input genomic DNA.

### 2.3. qRT-PCR

Quadriceps muscle was ground under liquid nitrogen with a mortar and pestle, homogenized with Trizol reagent per the manufacturer's protocol (Life Technologies, Foster City, CA) and column purified with a RNeasy kit (Qiagen, Valencia, CA). cDNA synthesis was carried out using M-MLV reverse transcriptase per the manufacturer's protocol (Promega, Madison, WI). qRT-PCR was performed on the ABI7900HT platform with SYBR Green PCR Master Mix (Applied Biosystems, Foster City, CA). Previously published primers targeted to* FLPGCC1α* and* NTPGC1α* were used [8].* Cyclophilin B* was used as the internal control. Data were analyzed using the standard curve method.

### 2.4. Determining −1N Subject Categories

Subjects were divided into two groups based on the results of our scanning qPCR, which was used to map the −1N position in the* PGC1α* promoter region. Subjects with a −1N positioned approximately between the −290 and −440 nt were designated to the upstream category (UP). Subjects with a −1 nucleosome positioned approximately between the −170 and −320 nt were designated to the downstream (DN) category.

### 2.5. Statistical Analyses

Previously measured anthropometric and cardiometabolic risk factors [14] and* FLPGC1α* and* NTPGC1α* mRNA expressions were averaged for each group, UP and DN. All data were analyzed by Student's *t*-test with *P* < 0.05 being considered significant using GraphPad Prism 4.0 software.

## 3. Results

### 3.1. Nucleosome Position and Splice Variant Expression

Several environmentally induced* PGC1α* splice variants are expressed in skeletal muscle, including* FLPGC1α* and* NTPGC1α* which have both been shown to act similarly to induce beneficial mitochondrial adaptations and improve metabolism in adipocytes [8, 18]. The −1N, which is important in determining gene transcription and expression and has recently been shown to determine splice variant expression [11], was mapped in the* PGC1α* promoter to determine its association with splice variant expression. Overlapping primer pairs were designed to span the* PGC1α* promoter region from approximately the −800 nt to the −100 nt (Figure 1(a)), and scanning qPCR was used to map the −1N position in* PGC1α*. Scanning qPCR gives a high resolution map of nucleosome position and occupancy at a specific genomic locus and is a common method for targeted nucleosome mapping [17, 19]. The −1 nucleosome showed phasing, ranging from the −440 nucleotide (nt) to the −170 nt (Figure 1(b)). Analysis of the phasing showed that individuals could be grouped based on the –1N being shifted either further upstream, away from the TSS (UP, *N* = 9), or closer downstream, toward the TSS (DN, *N* = 6) (Figure 1(b)).

**Figure 2:**
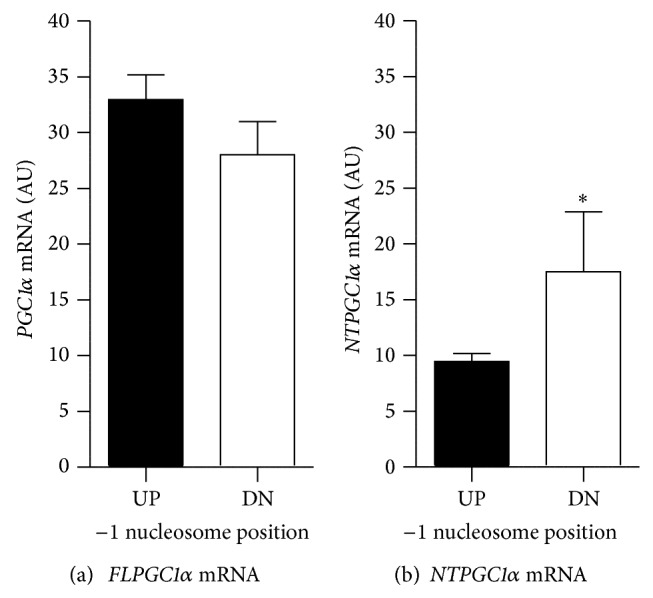
*PGC1α* gene expression. mRNA expression of* FLPGC1α* (a) and* NTPGC1α* (b) was measured by qRT-PCR in quadriceps muscle samples and mean ± SEM is shown as arbitrary units (AU) in upstream (UP, black) and downstream (DN, white) individuals. ∗ indicates significant difference between groups by Student's *t*-test with *P* < 0.05.

After grouping participant data based on the −1N position (Figure 1(b)), mRNA expressions of* FLPGC1α* and* NTPGC1α* were measured and analyzed. There was no significant difference in* FLPGC1α* mRNA expression between groups (*P* = 0.1746; Figure 2(a)). However, we observed a significant decrease in* NTPGC1α* mRNA expression in UP compared to DN (*P* = 0.0322; Figure 2(b)). These data suggest that nucleosome positioning in* PGC1α* may play a role in splice variant expression.

### 3.2. Nucleosome Position and CVD Risk Factors

**Figure 3:**
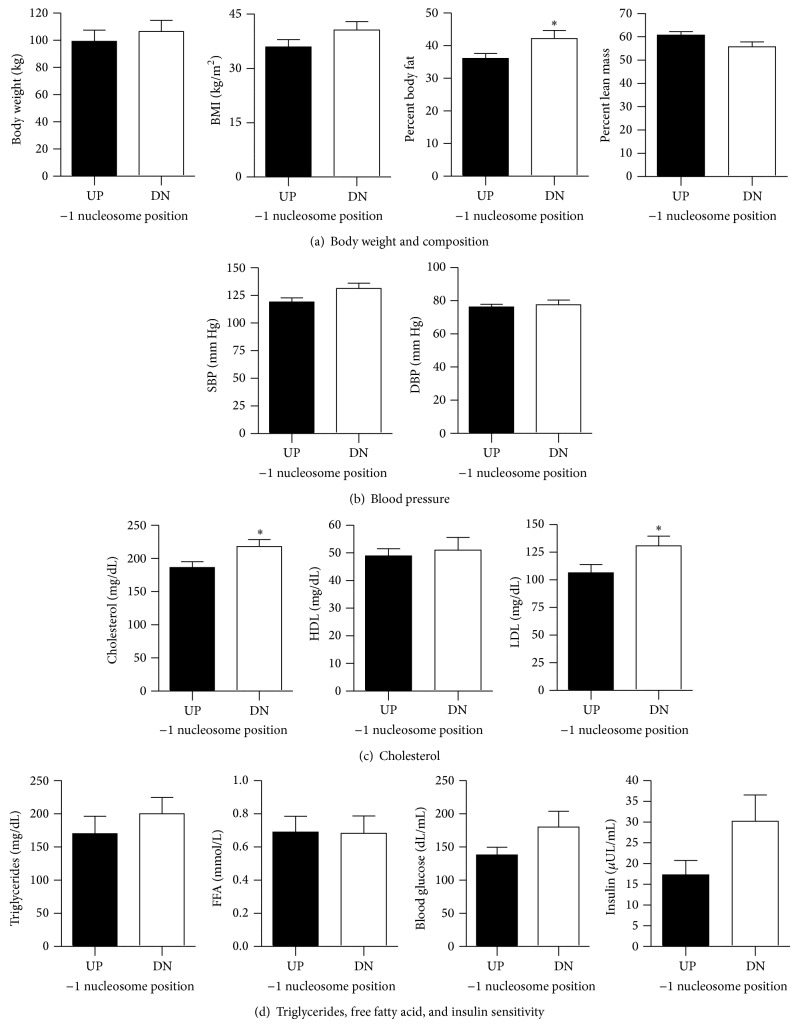
Anthropometric measures in individuals with alternate −1 nucleosome positioning within the* PGC1α* promoter. Individuals were divided into upstream (UP, black) and downstream (DN, white) groups and (a) body weight, body mass index (BMI), and percent body fat and lean mass, (b) systolic (SBP) and diastolic blood pressure (DBP), (c) total, high density lipoprotein (HDL), and low density lipoprotein (LDL) cholesterol, and (d) plasma triglycerides, free fatty acids (FFA), glucose, and insulin were analyzed by Student's *t*-test and are shown as mean ± SEM. ∗ indicates significance difference between groups with *P* < 0.05.

CVD risk is associated with obesity and T2D and individuals with lower expression of skeletal muscle* PGC1α* exhibit higher disease risk [20]. When subjects were divided into groups based on −1N position in* PGC1α*, no differences in body weight (Figure 3(a)) or age (UP 52.78 ± 2.91 y; DN 55.83 ± 3.05 y) existed between UP and DN. Interestingly, percent body fat was lower in UP compared with DN (*P* = 0.0455), although percentage of lean mass was not different (Figure 3(a)). BMI was not statistically significant between groups (Figure 3(a)). No significant differences in systolic (SBP) or diastolic blood pressure (DBP) were evident (Figure 3(b)). In UP, total serum cholesterol (*P* < 0.04) and low density lipoprotein (LDL; *P* < 0.04) cholesterol were lower, and there was no difference in high density lipoprotein (HDL) cholesterol between groups (Figure 3(c)). Serum triglycerides, free fatty acids (FFA), fasting blood glucose, and insulin levels were not different between groups (Figure 3(d)). These data show that individuals with a −1N positioned proximal to the TSS in the* PGC1α* promoter and with higher levels of* NTPGC1α* exhibit increased CVD risk as assessed by adiposity, total cholesterol, and LDL cholesterol.

## 4. Discussion

We mapped the −1N within the* PGC1α* promoter region to provide insight into differential epigenetic regulation of* PGC1α*, including* FLPGC1α* and* NTPGC1α* splice variant expression, in overweight/obese individuals with T2D and to provide evidence that alterations in* PGC1α*  −1N position and splice variant expression are associated with differential CVD risk [10]. Our results provide evidence that proximal positioning of the −1N in* PGC1α* is associated with increased CVD risk and increased* NTPGC1α* expression. Interestingly, we found that, in overweight/obese individuals with T2D exhibiting higher adverse CVD risk, the −1N was positioned over a regulatory epigenetic site in the* PGC1α* promoter [7], which may be dependent on nucleosome positioning [10]. The −1N was associated with the degree of adiposity but not fasting insulin or glucose levels, with those individuals exhibiting a −1N proximal to the TSS, over the regulatory epigenetic site, being more obese and having higher levels of total and LDL cholesterol. These data suggest that the chromatin structure of* PGC1α* [7, 14] is related to the degree of overweight/obesity and obesity-associated CVD risk in individuals with T2D. Indeed, others have noted that epigenetic regulation of skeletal muscle* PGC1α* resulting in decreased gene expression leads to a reduction in skeletal muscle mitochondrial number and decreased expression of* PGC1α* target genes in association with disease state, specifically insulin resistance and T2D [7, 21].

Although −1N position was not associated with alterations in* FLPGC1α* expression, those individuals with the −1N more proximal to the TSS showed an increase in* NTPGC1α* expression in addition to increased CVD risk. Recent research indicates a role of the −1N in regulating transcript processing via mRNA splicing [11]. Here, we found differential −1N in* PGC1α* predicted* NTPGC1α* but not* FLPGC1α* expression in skeletal muscle, with a significant increase in* NTPGC1α* in the skeletal muscle of DN individuals who had their −1N positioned proximal to the TSS. These observed differences in splice variant expression between UP and DN groups in the present study suggest that the −1N may regulate* PGC1α* splicing and variant expression. Although the mechanism of this regulation is yet to be explored, it is possible that phasing of the −1N may determine periodicity of downstream nucleosomes or decrease transcription elongation rate, leading to intron 6 inclusion and* NTPGC1α* expression [8]. Importantly,* NTPGC1α* has been shown to translocate to the nucleus in adipose tissue, where it acts in a similar manner to* FLPGC1α* to regulate nuclear-encoded mitochondrial gene expression [8, 22, 23]. Splice variant expression may also be important in determining mitochondrial function and number in skeletal muscle [9, 24–26], and* NTPGC1α* expression may be increased to compensate for lack of change in* FLPGC1α* [18]. Indeed, it has recently been reported that upregulation of* NTPGC1α* in myotubes increases glucose transporter and mitochondrial gene expression, which may account partially for the similarities between* FLPGC1α* and* NTPGC1α* in their insulin sensitizing effects [27]. Although it is possible that* NTPGC1α* has differential functions in various tissues, we speculate that in the present study* NTPGC1α* is upregulated to compensate for lack of change in* FLPGC1α* in the obese and diabetic state. However, the magnitude of* NTPGC1α* increase may not be adequate or sufficient to ameliorate obesity-associated metabolic dysfunction and CVD risk in this population. The lack of data on differential expression of* PGC1α* target genes in the present study is a limitation and the focus of future studies on these particular differences in obese and diabetic individuals will provide insight into the molecular mechanisms linking* PGC1α* DNA methylation to −1N positioning, the role of epigenetics in determining splice variant expression, and the associations of splice variants' expression to* PGC1α* target gene activation and disease state. Additionally, further analysis of splice variant expression levels should be conducted in lean versus overweight/obese individuals as well as other diseased and nondiseased populations to determine a minimum level of* FL*- or* NTPGC1α* that is sufficient to decrease CVD risk.

## 5. Conclusions

Our data revealed that downstream −1N in the* PGC1α* promoter is associated with higher adiposity and adverse health risk, whereas those individuals with an upstream −1N had lower adiposity and obesity-related risk for CVD. Our data suggest that −1N positioning may be a potential epigenetic mechanism that regulates* NTPGC1α* splice variant expression, and this variant expression is linked to CVD risk in overweight/obese individuals with T2D.

## Figures and Tables

**Table 1:** Scanning qPCR primer pair sequences for *PGC1α*.

Primer pair	Forward primer sequence	Reverse primer sequence
1	AGAGCAGCAGCGACTGTAT	TAC CAG CTC CCG AAG AGT TG
2	CAA CTC TTC GGG AGC TGG TA	TGA GGG AGT GTT TGA AAG CG
3	CGC TTT CAA ACA CTC CCT CA	GCA AAG CTC CCT GTT TCA TGA C
4	GTC ATG AAA CAG GGA GCT TTG C	GAGGCTTCAAGCATCATGCT

## Abbreviations

−1N: −1 nucleosome
BMI: Body mass index
CVD: Cardiovascular disease
DN: Downstream
DNMT3b: DNA methyltransferase 3b
DBP: Diastolic blood pressure
ERR: Estrogen related receptor alpha
FFA: Free fatty acids
HART-D: Health Benefits of Aerobic and Resistance Training in Type 2 Diabetes
HDL: High density lipoprotein
*FLPGC1α*: Full length peroxisome proliferator-activated receptor gamma coactivator 1 alpha
LDL: Low density lipoprotein
MNase: Micrococcal nuclease
Nrf1: Nuclear receptor factor 1
*NTPGC1α*: N truncated peroxisome proliferator-activated receptor gamma coactivator 1 alpha
*PGC1α*: Peroxisome proliferator-activated receptor gamma coactivator 1 alpha
PPAR: Peroxisome proliferator-activated receptor
SBP: Systolic blood pressure
T2D: Type 2 diabetes
TSS: Transcriptional start site
UP: Upstream.
